# Use of Real-Time PCR for *Chlamydia psittaci* Detection in Human Specimens During an Outbreak of Psittacosis — Georgia and Virginia, 2018

**DOI:** 10.15585/mmwr.mm7014a1

**Published:** 2021-04-09

**Authors:** Olivia L. McGovern, Miwako Kobayashi, Kelly A. Shaw, Christine Szablewski, Julie Gabel, Caroline Holsinger, Cherie Drenzek, Skyler Brennan, Jennifer Milucky, Jennifer L. Farrar, Bernard J. Wolff, Alvaro J. Benitez, Kathleen A. Thurman, Maureen H. Diaz, Jonas M. Winchell, Stephanie Schrag

**Affiliations:** ^1^Epidemic Intelligence Service, CDC; ^2^Division of Bacterial Diseases, National Center for Immunization and Respiratory Diseases, CDC; ^3^Virginia Department of Health; ^4^Georgia Department of Public Health.

Psittacosis is typically a mild febrile respiratory illness caused by infection with the bacterium *Chlamydia psittaci* and usually transmitted to humans by infected birds (*1*). On average, 11 psittacosis cases per year were reported in the United States during 2000–2017. During August–October 2018, the largest U.S. psittacosis outbreak in 30 years (82 cases identified*) occurred in two poultry slaughter plants, one each in Virginia and Georgia, that shared source farms (*2*). CDC used *C. psittaci* real-time polymerase chain reaction (PCR) to test 54 human specimens from this outbreak. This was the largest number of human specimens from a single outbreak ever tested for *C. psittaci* using real-time PCR, which is faster and more sensitive than commercially available serologic tests. This represented a rare opportunity to assess the utility of multiple specimen types for real-time PCR detection of *C. psittaci*. *C. psittaci* was detected more frequently in lower respiratory specimens (59% [10 of 17]) and stool (four of five) than in upper respiratory specimens (7% [two of 28]). Among six patients with sputum and nasopharyngeal swabs tested, *C. psittaci* was detected only in sputum in five patients. Cycle threshold (Ct) values suggested bacterial load was higher in lower respiratory specimens than in nasopharyngeal swabs. These findings support prioritizing lower respiratory specimens for real-time PCR detection of *C. psittaci*. Stool specimens might also have utility for diagnosis of psittacosis.

Use of primarily serologic tests for laboratory confirmation of psittacosis might contribute to underdiagnosis. The most commonly available diagnostic tests for psittacosis are commercially available serologic tests. These tests have poor specificity and require testing of paired sera collected weeks apart, delaying or preventing confirmation of clinical diagnoses (*3*). Real-time PCR assays are sensitive, specific, and can be performed in hours. However, in the United States, real-time PCR assays for diagnosis of psittacosis using human respiratory specimens are currently available only at CDC (*4*).

During August 31–September 12, 2018, the Virginia Department of Health and Georgia Department of Public Health were each notified of a cluster of patients hospitalized with symptoms consistent with psittacosis; all worked in one of two chicken slaughter plants that shared source farms, one in Virginia and one in Georgia (*2*). In Virginia and Georgia, local and state public health officials conducted active case finding to identify illness consistent with psittacosis in persons who worked at one of the plants during August–September 2018. Workers were classified as having probable or confirmed cases of illness based on case definitions. Probable cases were identified based on symptoms and epidemiologic exposures, and confirmed cases were identified based on detection of *C. psittaci* by real-time PCR in at least one clinical specimen.^†^ Clinical specimens for *C. psittaci* testing were collected from workers seeking medical care, at the discretion of local clinicians, and sent to CDC for real-time PCR testing; no diagnostic testing for psittacosis was performed elsewhere. CDC recommended collection of lower respiratory specimens whenever possible, but all available specimens were accepted and tested.^§^ Testing was performed in triplicate using extracted total nucleic acid^¶^ and oligonucleotides targeting the *C. psittaci* locus tag CPSIT_RS01985 on an ABI 7500 real-time PCR system (*4*,*5*). A specimen was considered positive for *C. psittaci* if amplification of the CPSIT_RS01985 locus tag was detected. Patient demographic, clinical, and specimen characteristics among the subset of workers who submitted specimens to CDC were determined by patient interview, medical chart abstraction, and laboratory record review.

Frequency of *C. psittaci* detection among all specimens and mean Ct value among *C. psittaci*–positive specimens were evaluated for each specimen type. Frequency of demographic and clinical characteristics were determined for probable and confirmed cases. All analyses were performed using SAS (version 9.4; SAS Institute). This activity was reviewed by CDC and was conducted consistent with applicable federal law and CDC policy.**

Among 82 ill workers identified by Virginia and Georgia departments of health, 33 (40%) submitted a total of 54 specimens for real-time PCR testing. Thirteen of the 33 (39%) workers tested had confirmed cases of psittacosis, and 20 (61%) had probable cases of psittacosis (Table 1). Compared with probable cases, confirmed cases tended to be identified among older patients. A higher proportion of persons with confirmed cases were male, had a diagnosis of pneumonia, were hospitalized, or required intensive care unit admission. The most commonly submitted specimen type was nasopharyngeal swab (18 of 20 [90%] of probable and eight of 13 confirmed cases). Nasopharyngeal swab specimens only were submitted by 13 (65%) of 20 persons with probable cases and one of 13 persons with confirmed cases. Lower respiratory specimens were submitted by six (30%) of 20 persons with probable cases and most (10 of 13) persons with confirmed cases. Timing of specimen collection relative to illness onset was similar for persons with probable (mean = 7 days; range = 2−13 days) and confirmed (mean = 6 days; range = 1−14 days) cases. Most patients received antibiotic treatment before or on the same day as specimen collection for *C. psittaci* testing (Table 1).

**TABLE 1:** Characteristics of persons with probable and confirmed cases associated with a psittacosis outbreak — Georgia and Virginia, 2018

Characteristic	Cases, no. (%)	
	Probable* (n = 20)	Confirmed^†^ (n = 13)
Age, yrs, mean (range)	36 (22–55)	48 (29–57)
Male	11 (55)	10 (77)
**Clinical characteristic**		
Physician-diagnosed pneumonia	15 (75)	12 (92)
Hospitalized	12 (60)	11 (85)
Admitted to intensive care unit	0 (—)	2 (15)
**Specimen type submitted^§^**		
**Upper respiratory**		
Nasopharyngeal swab	18 (90)	8 (62)
Oropharyngeal swab	1 (5)	0 (—)
**Lower respiratory**		
Sputum	6 (30)	8 (62)
Bronchoalveolar lavage	0 (—)	2 (15)
**Nonrespiratory**		
Stool	0 (—)	5 (38)
Blood	2 (10)	0 (—)
Cerebrospinal fluid	1 (5)	0 (—)
**Only nasopharyngeal specimens submitted**	12 (60)	1 (8)
**Days from illness onset to specimen collection, mean (range)**	7 (2–13)	6 (1–14)
**Antibiotic treatment relative to specimen collection^¶^**		
Before	9 (45)	10 (77)
Same day	8 (40)	3 (23)
After	3 (15)	0 (—)

**Abbreviation:** PCR = polymerase chain reaction.

* Cases of illness in persons who worked at the Virginia plant during August 1–September 7, 2018, or at the Georgia plant during August 13–September 28, 2018, and had physician-diagnosed pneumonia, fever, or chills with two or more of the following: headache, cough, or muscle aches. All probable cases in this analysis were real-time PCR–negative for *Chlamydia psittaci*.

**^†^** Cases of illness in persons who had real-time PCR detection of *C. psittaci* in at least one clinical specimen with or without meeting the probable case definition.

^§^ A total of 18 patients submitted multiple specimens; therefore, the sum of patients submitting each specimen type exceeds 33.

^¶^ The percentage of patients with doxycycline or a macrolide antibiotic treatment, first- and second-line antibiotics against psittacosis, initiated before, on the same day as, or after specimens for *C. psittaci* testing were collected. It could not be distinguished whether antibiotic treatment occurred before or after specimen collection for patients with antibiotic treatment initiation and specimen collection occurring on the same day.

*C. psittaci* was most commonly detected in stool (four of five specimens) and lower respiratory specimens of bronchoalveolar lavage (two of two) and sputum (eight of 15), and less frequently in upper respiratory specimens of nasopharyngeal swabs (two [7%] of 27) and oropharyngeal swabs (zero of one) (Table 2). Among *C. psittaci*–positive specimens, lower respiratory specimens had lower Ct values (mean = 29; range = 26–31), indicating higher bacterial load, than did nasopharyngeal swabs (Ct values 31 and 33) and stool specimens (mean = 34; range = 32–37).

**TABLE 2:** Real-time PCR test results, by specimen type, for all specimens tested in association with a psittacosis outbreak — Georgia and Virginia, 2018

Specimen type	No. of specimens tested	*C. psittaci*–positive specimens, no. (%)	Ct value among *C. psittaci*–positive specimens, mean (range)
**Upper respiratory**			32 (31–33)*
Nasopharyngeal swab	27	2 (7)	
Oropharyngeal swab	1	0 (—)	
**Lower respiratory**			29 (26–31)
Sputum	15	8 (53)	
Bronchoalveolar lavage	2	2 (100)	
**Nonrespiratory**			34 (32–37)**^†^**
Stool	5	4 (80)	
Blood	3	0 (—)	
Cerebrospinal fluid	1	0 (—)	

**Abbreviations:** *C. psittaci* = *Chlamydia psittaci*; Ct = cycle threshold; PCR = polymerase chain reaction.

* Data are from two nasopharyngeal swabs with Ct values 31 and 33.

**^†^** Data are only from stool specimens because there were no *C. psittaci*–positive blood or cerebrospinal fluid specimens.

Among 13 patients with confirmed psittacosis, nine submitted multiple specimen types, allowing comparison of *C. psittaci* detection by specimen type (Table 3). Six patients had nasopharyngeal swab and sputum specimens tested; all sputa tested positive for *C. psittaci*, but only one nasopharyngeal swab tested positive. Three patients submitted stool and sputum specimens; all three sputum specimens tested positive for *C. psittaci,* and two stool specimens tested positive. *C. psittaci* was also detected in stool specimens of two patients with *C. psittaci*–negative nasopharyngeal swabs, including patient A, who had positive sputum and stool specimens but a negative nasopharyngeal swab. Ct values were lower in sputum than in stool specimens or nasopharyngeal swabs from the same patient (patients A, F, and G).

**TABLE 3:** Clinical findings, hospitalization status, and real-time PCR test results in patients* with confirmed psittacosis cases (n = 13) associated with a psittacosis outbreak — Georgia and Virginia, 2018

Clinical findings and hospitalization status				Real-time PCR result by specimen type (Ct value)^†^			
**Patient**	**Pneumonia^§^**	**Hospitalized**	**Admitted to ICU**	**Sputum**	**BAL**	**NP swab**	**Stool**
A	Yes	Yes	No	Pos (30)	—^¶^	Neg	Pos (37)
B	Yes	Yes	No	Pos (30)	—	Neg	—
C	Yes	No	No	Pos (28)	—	Neg	—
D**	Yes	Yes	No	Pos (26)	—	Neg	—
E	Yes	Yes	No	Pos (26)	—	Neg	—
F^††^	Yes	Yes	Yes	Pos (27)	—	Pos (33)	—
G	Yes	Yes	No	Pos (28)	—	—	Pos (32)
H	Yes	Yes	No	Pos (30)	—	—	Neg
I	Yes	Yes	No	—	Pos (31)	—	—
J	Yes	Yes	Yes	—	Pos (30)	—	—
K^§§^	No	Yes	No	—	—	Pos/Neg (31)	—
L	Yes	Yes	No	—	—	Neg	Pos (38)
M	Yes	No	No	—	—	—	Pos (32)

**Abbreviations:** BAL = bronchoalveolar lavage; Ct = cycle threshold; ICU = intensive care unit; Neg = negative; NP = nasopharyngeal; PCR = polymerase chain reaction; Pos = positive.

* All specimens associated with an individual patient were collected on the same day unless otherwise noted. For example, sputum, NP swab, and stool specimens for patient A were all collected on the same day, but specimens from patient A were not necessarily collected on the same day as were those from patient B.

^†^ ”Pos” indicates *Chlamydia psittaci* was detected. “Neg” indicates *C. psittaci* was not detected. Ct values represent the real-time PCR amplification cycle at which CPSIT_RS01985 amplification was first detected. Ct values are displayed only for *C. psittaci–*positive specimens, because CPSIT_RS01985 was not detected in *C. psittaci*–negative specimens.

^§^ Radiograph-confirmed pneumonia.

^¶^ Dashes indicate specimen type was not submitted to CDC.

** Sputum was collected 3 days after the NP swab.

^††^ Sputum was collected 1 day after the NP swab.

^§§^ Two NP swabs were tested; one tested positive and one tested negative. The negative NP swab was collected 2 days after the positive NP swab.

## Discussion

This was the largest U.S. psittacosis outbreak in 30 years, the first U.S. outbreak in which human specimens were tested exclusively by real-time PCR, and the first that included testing of stool specimens. In this outbreak investigation, as in other published studies with real-time PCR–based detection of *C. psittaci* (*3*), *C. psittaci* was more frequently detected in lower respiratory specimens than in upper respiratory specimens, as reflected by detection in sputum but not nasopharyngeal swabs in five confirmed cases. Ct values in *C. psittaci*–positive specimens also suggest that bacterial load is higher in lower respiratory specimens than in nasopharyngeal swab specimens. This analysis suggests that lower respiratory specimens are more useful than upper respiratory specimens for *C. psittaci* detection by real-time PCR. Although submission of lower respiratory specimens is encouraged, upper respiratory specimens are easier to collect, which could explain why nasopharyngeal swab was the most frequently submitted specimen type. Given that 60% of patients with probable cases submitted only nasopharyngeal swab specimens, *C. psittaci* might have been underdetected in this outbreak; it is possible that more confirmed cases would have been detected if lower respiratory specimens were collected for testing in these patients.

Although only five stool specimens were tested in this outbreak, the frequency of *C. psittaci* detection in stool specimens was high, and in two patients *C. psittaci* was detected in stool and sputum specimens. Gastrointestinal symptoms have been reported among psittacosis patients (*6*–*8*), and three of four patients with *C. psittaci* detection in stool specimens in this investigation also had gastrointestinal symptoms. However, whether these symptoms correspond with detection of *C. psittaci* in stool specimens has not been documented. Additional studies are needed to validate whether detection of *C. psittaci* DNA in stool specimens alone indicates presence of infectious bacteria in humans. Nonetheless, this investigation provides promising evidence that stool specimens might have utility for diagnosis of psittacosis using real-time PCR.

The findings in this report are subject to at least two limitations. First, specimens were not systematically collected and were available from only a subset of patients. Because of this, the sample size overall and per specimen type was small. The small sample size limited ability to assess how severity of illness and antibiotic treatment affect *C. psittaci* detection for each specimen type. Second, although lower respiratory specimen collection was encouraged, psittacosis is characterized by dry cough, and lower respiratory specimens are difficult to obtain from mildly ill patients unless sputum collection is induced. Patients with severe illness, who likely also have higher bacterial load, might have been more likely to submit lower respiratory specimens.

Many factors influence *C. psittaci* detection in human clinical specimens; these include specimen type, timing of collection relative to illness onset and treatment with tetracycline or macrolide antibiotics, and severity of illness. Collecting information about these factors and systematic, serial testing of multiple specimen types from suspected cases might help inform optimal conditions for *C. psittaci* detection using real-time PCR. Public health professionals and health care providers should be aware that *C. psittaci* might not be detected if nasopharyngeal swab specimens alone are tested and that collection of respiratory specimens from multiple sites can improve detection by real-time PCR. Although lower respiratory specimens collected shortly after symptom onset might have the highest yield to diagnose psittacosis using real-time PCR, stool specimens might also have utility for diagnosis of psittacosis.

**Summary**

**What is already known about this topic?**

Real-time polymerase chain reaction (PCR) testing for *Chlamydia psittaci*, the bacterium that causes psittacosis, is faster and more specific than widely available serologic tests. However, the utility of diverse specimen types for *C. psittaci* detection by real-time PCR is unknown.

**What is added by this report?**

During a large psittacosis outbreak in 2018, *C. psittaci* was most frequently detected in lower respiratory and stool specimens using real-time PCR.

**What are the implications for public health practice?**

It is important for clinicians and public health professionals to prioritize collection of lower respiratory specimens for *C. psittaci* real-time PCR testing. Findings of this outbreak investigation provide preliminary evidence that stool specimens might have utility for diagnosis of psittacosis.

## Footnotes

* Although 80 cases had been reported previously, a retrospective review of CDC and medical records and discussion with state partners led to an increase of the case count to 82. During September 19–20, 2018, a health hazard evaluation was conducted at the Virginia plant by the National Institute for Occupational Safety and Health at the request of the U.S. Department of Agriculture Food Safety and Inspection Service. Recommendations to reduce worker risk included repositioning cooling fans, ensuring proper function of evisceration tools, and other changes to workplace practices to reduce bacterial contamination and aerosolization.

† Among those who worked at the Virginia plant during August 1–September 7, 2018, or at the Georgia plant during August 13–September 28, 2018, persons with probable cases had physician-diagnosed pneumonia, fever, or chills with two or more of the following: headache, cough, or muscle aches. Patients with confirmed cases had real-time PCR detection of *C. psittaci* in at least one clinical specimen with or without meeting the probable case definition.

§ A total of seven specimen types were tested, including upper respiratory specimens (nasopharyngeal swab or oropharyngeal swab), lower respiratory specimens (sputum or bronchoalveolar lavage), and nonrespiratory specimens (stool, blood, and cerebrospinal fluid).

¶ Total nucleic acid was extracted from all specimens using a MagNA Pure Compact instrument (Roche Diagnostics) with the Roche Total Nucleic Acid Isolation Kit I according to manufacturer instructions; elution volume was 100 *μ*L. Stool, whole blood, and sputum specimens were preprocessed before nucleic acid extraction. For stool specimens, 200 mg of stool were first resuspended in 2 mL of phosphate buffered saline (PBS). Then, 400 *μ*L of the resuspended stool specimen was inactivated by combining with 400 *μ*L MagNA Pure Bacteria Lysis Buffer and 40 *μ*L Proteinase K (25 mg/mL; Thermo Fisher Scientific). Inactivated specimens were subject to mechanical lysis by bead-beat with 0.5 mm glass beads using a Precellys 24 Homogenizer (Bertin Corp.) at 5,000 rpm for 60 seconds twice with a 5-second hold in between. The specimen was then centrifuged at 10,000×g for 1 minute, and 700 *μ*L of supernatant was used for total nucleic acid extraction. Whole blood was not resuspended with PBS, but otherwise underwent the same preprocessing as stool. For sputum specimens, 300 *μ*L of the specimen was combined with 300 *μ*L of 1,4-Dithiothreitol (12.5mM; Fisher Scientific) and incubated at room temperature for 1 hour.

** 45 C.F.R. part 46, 21 C.F.R. part 56; 42 U.S.C. Sect. 241(d); 5 U.S.C. Sect. 552a; 44 U.S.C. Sect. 3501 et seq.
